# The Role of Existential Concerns in the Individual’s Decisions regarding COVID-19 Vaccine Uptake: A Survey among Non-Vaccinated Italian Adults during the Third Wave of the Pandemic

**DOI:** 10.3390/vaccines10071079

**Published:** 2022-07-05

**Authors:** Vittoria Franchina, Rubinia Celeste Bonfanti, Gianluca Lo Coco, Laura Salerno

**Affiliations:** 1 Department of Psychology, University of Salzburg, 5020 Salzburg, Austria; vittoria.franchina923005@gmail.com; 2 Department of Psychology, Educational Science and Human Movement, University of Palermo, Viale delle Scienze, Edificio 15, 90128 Palermo, Italy; rubiniaceleste.bonfanti@unipa.it (R.C.B.); gianluca.lococo@unipa.it (G.L.C.)

**Keywords:** vaccine hesitancy, existential concerns, basic psychological needs, attitudes, COVID-19, Pfizer-BioNTech vaccine, AstraZeneca vaccine

## Abstract

Recent studies have suggested that health constructs embraced by the Terror Management Theory (TMT) and the Basic Psychological Needs Theory (BPNT) may drive individuals’ COVID-19 health-related decisions. This study examines the relationships between existential concerns (ECs; within the TMT), basic psychological needs (BPNs; within the BPNT) and COVID-19 vaccine hesitancy (VH), as well as the mediating role of negative attitudes toward COVID-19 vaccines. A cross-sectional survey was carried out from April to May 2021 on a sample of two hundred and eighty-seven adults (M_age_ = 36.04 ± 12.07; 59.9% females). Participants provided information regarding existential concerns, basic psychological needs, attitudes toward COVID-19 vaccines and vaccine hesitancy for Pfizer-BioNTech and AstraZeneca vaccines separately. Higher vaccine hesitancy (32.1%) and vaccine resistance (32.8%) rates were found for AstraZeneca than for Pfizer-BioNTech COVID-19 vaccine (22.3% and 10.1%, respectively). Structural equation modeling showed that existential concerns were related to Pfizer-BioNTech and AstraZeneca vaccine hesitancy both directly and indirectly through negative attitudes toward potential side effects of COVID-19 vaccines. The findings of the study confirm that the TMT is efficient in explaining COVID-19 vaccine hesitancy. Targeted efforts are needed to increase the acceptance of COVID-19 vaccines.

## 1. Introduction

Since December 2019, starting in Wuhan, China, and continuing for more than two years now, a cluster of acute respiratory illnesses, known as coronavirus disease (COVID-19), has spread globally. On 30 January 2020, the World Health Organization (WHO) [1] called the COVID-19 outbreak a “public health emergency of international concern”. This occupied the headlines in the media all around the world and on 11 March WHO declared this to be a pandemic. Starting in the spring of 2020, several countries had to use restrictive, preventive measures to ensure physical distancing and slow down the spread of the new virus. Consequently, life circumstances changed drastically, and several aspects of people’s everyday lives went through major changes.

Since COVID-19 vaccination became available, several misconceptions have globally spread among the general population (e.g., severe side effects, use of fetal tissue in the development of the vaccine, presence of microchips inside the vaccine), probably due to the rapid development of the vaccines [2]. In Italy, vaccination became available on the 27 December 2020, but by April 2021 only 3,500,000 adults had received the COVID-19 vaccine. Although there is considerable evidence regarding the necessity of COVID-19 inoculations for both one’s own health and that of society, almost one Italian in five believed that vaccines were harmful and often reported no faith in scientists [3]. It is likely that psychological factors may have played a central role in determining vaccine hesitancy among the Italian adult population, in the face of a good level of knowledge regarding COVID-19 and its prevention [4]. Thus, initial research efforts focused on examining the underlying mechanisms and factors that may be linked to COVID-19 vaccine hesitancy (VH).

The present study investigates whether basic psychological needs (BPNs) and existential concerns (ECs) might lead to greater negative attitudes toward COVID-19 vaccines, which, in turn, might lead to VH.

### 1.1. BPNs and VH

The Basic Psychological Needs Theory (BPNT), is one of the six mini-theories of Self-Determination Theory (SDT) [5,6]. BPNT posits the existence of three BPNs: relatedness, competence and autonomy. Relatedness satisfaction involves the experience of intimate connection with others [7], whereas relatedness frustration means experiencing social exclusion and loneliness. Competence satisfaction refers to feeling capable of achieving desired outcomes [7,8], whereas competence frustration means the experiencing of failure and doubts regarding self-efficacy. Autonomy regards feelings of self-determination and total willingness when pursuing an activity, whereas autonomy frustration means feeling controlled through externally imposed influences [5,9]. BPNT argues that the satisfaction of these BPNs is necessary for humans’ psychological wellbeing. When these needs are frustrated, defensiveness and ill-being may arise [10,11,12]. Moreover, the satisfaction of these needs has been linked to better health choices [13,14,15,16]; conversely, the experience of need frustration might lead people to react with oppositional defiance, thereby leading to the opposite of what is requested and escaping from feeling controlled [12,17].

Social distancing and restrictions due to the COVID-19 pandemic severely frustrated individuals’ BPNs. More specifically, since spring 2020, governments have taken strict measures with the intention of slowing the contagion rate. In Italy, measures were primarily focused on social distancing and lockdown; educational institutions and service facilities were closed (except for pharmacies and food stores); public gatherings were forbidden; when allowed to leave home, people could not gather in more than a limited number. Unfortunately, previous studies showed that these restrictions might have affected individuals’ perceptions of their inability to satisfy psychological needs or might directly frustrate them [18,19,20]. For example, people were not free to socialize, nor to easily achieve their own desired goals as well as being generally restricted in their own autonomy, i.e., being forced to wear a face mask [19,21,22,23].

Given the influence of BPNs in health decision processes [13,14,15,16] and the frustration of individuals’ BPNs due to the pandemic, it is likely that frustration of BPNs might be involved in the mechanism of refusing the COVID-19 vaccine. Previous studies [17,24,25] showed that frustration of one’s BPNs has direct consequences, such as loss of motivation, disengagement, and unhealthy, or even reactant behavior. Reactant behavior is the resistance to attempts at persuasion or external influences (such as the perceived pressure of getting COVID-19 vaccines), and occurs when one is striving for autonomy, competence, and freedom [26,27,28]. In addition, loneliness, which occurs when the relatedness need is frustrated, is also often related to reactant and non-medically compliant behavior [27,29,30]. To our knowledge, no previous studies have examined the role of BPNs in predicting individual willingness to get vaccinated against COVID-19 and the present study aims to fill this gap in the literature.

### 1.2. ECs and VH

Significant global events, such as the COVID-19 pandemic, might provoke thoughts of death. Moreover, in the last few years, social isolation, growing unemployment, and restriction of freedom have triggered awareness of death and have affected people’s approach to meanings of life [31]. This has led to an increase in ECs, which, in turn, might have significant effects on people’s attitudes and health-related behavior.

Recent studies have suggested the adoption of the Terror Management Theory (TMT) to predict COVID-19 vaccine intentions. More specifically, within the TMT, previous studies suggested that COVID-19 is linked to increased existential anxieties and concerns, which may, subsequently, affect COVID-19 health-related decisions [32,33]. Already before the COVID-19 pandemic, some studies [34] showed that individuals may engage in behavior harmful to one’s health, such as smoking and acquiring a sun-tan, in the wake of intimations of mortality, whenever this behavior confirmed one’s personal cultural worldviews and affected one’s self-esteem. Furthermore, the General Process Model of Threat and Defense Model (GPM) [35] goes further, including not only the threat of mortality, but also other existential omens, such as meaninglessness, loneliness, uncertainty, loss of control, proposing a common, underlying, motivational process for people’s reactions to threats. Following various kinds of threats (such as the ones triggered by COVID-pandemic and restrictions), individuals might focus more strongly on their ideals, social identities and worldviews to recover from this anxiety; this might argue for strongly supporting all types of belief in favor of, or against, COVID-19 vaccines. Regarding the willingness to be vaccinated against COVID-19, a recent study [36] showed a positive relationship between fear of COVID-19 and intention to get vaccinated; however, when this fear was associated with high levels of ECs through conspiracy beliefs, the intention to get vaccinated diminished. Bodner et al. [37] also showed that death anxiety was positively associated with COVID-19 vaccination anxiety. Moreover, Simione et al. [38] found that death anxiety has both a positive direct effect on COVID-19 vaccine propensity (i.e., increasing the propensity to get vaccinated) as well as an indirect effect through belief in conspiracy (i.e., reducing the vaccine propensity by increasing belief in conspiracy theories). The authors [38] suggested that both faith in the vaccine and conspiracy beliefs may be viewed as a form of mitigation of existential fears and concerns, in the wake of death anxiety. The present study aims to add more new knowledge in this field by examining a broader construct of existential concerns (i.e., general existential anxiety, death anxiety and avoidance) in relation to different types of COVID-19 vaccines.

Following this line of thought, it is likely that people experiencing higher ECs, and already having negative attitudes towards vaccines, might also be more hesitant in getting vaccinated; this is because ECs might lead them to amplify their already negative attitudes and beliefs, as a defense against anxiety.

### 1.3. The Present Study

The study aims to examine the relationships between ECs (within the TMT), BPNs (within the BPNT) and VH, and the mediating effect of negative attitudes (i.e., efficacy and potential side effects) toward COVID-19 vaccines (both Pfizer-BioNTech and AstraZeneca).

It has been hypothesized that a higher presence of ECs and BPNs would increase negative attitudes toward COVID-19 vaccines (about both their efficacy and their potential side effects) and, in turn, lead to increased VH.

Moreover, since previous studies [39] showed that VH is higher for viral vector (e.g., the AstraZeneca vaccine) than mRNA (e.g., the Pfizer-BioNTech vaccine) COVID-19 vaccines, and that factors related to viral vector VH were partially different from those related to mRNA VH, in the current study we examine VH separately for Pfizer-BioNTech and AstraZeneca vaccines. It has been hypothesized that the relationships between ECs, BPNs, attitudes toward COVID-19 vaccines and VH should be stronger for the AstraZeneca vaccine.

## 2. Materials and Methods

### 2.1. Participants

A total of 396 participants originally took part in the study: Italian citizens who were aged older than 18 were recruited via the crowdsourcing platform Clickworker. The platform’s crowd consists of a large population of active workers. As we were interested in participants’ attitudes toward COVID-19 vaccines and vaccine hesitancy, 109 participants were subsequently excluded because they had already received the first dose of a COVID-19 vaccine. Two-hundred and eighty-seven adults (age: M = 36.04, SD = 12.07) from different Italian regions (Northern, Central and Southern Italy) completed the survey. Table 1 provides a detailed description of participant demographics and health-related characteristics.

### 2.2. Measures

#### 2.2.1. ECs

Existential concerns were evaluated with the Existential Concerns Questionnaire (ECQ) [40], a 22-item measure. It is composed of the following three subscales: (a) General existential anxiety (12 items; e.g., “*The question of whether life has meaning makes me anxious*”), (b) Death anxiety (6 items; e.g., “*It makes me anxious that my life is passing by*”) and (c) Avoidance (4 items; e.g., “*I try to push away the thought that life will end*”). Each item was rated on a five-point Likert scale from 1 (totally disagree) to 5 (totally agree). Higher scores indicate a greater degree of fear brought about by the main threats to human existence, such as death and fundamental loneliness. All subscales showed good internal consistency (mean inter-item correlation was 0.503, 0.581 and 0.474 for General existential anxiety, Death anxiety and Avoidance subscales, respectively).

#### 2.2.2. BPNs

Basic Psychological Needs were evaluated with three subscales from the Basic Psychological Need Satisfaction Scales [41]. The following subscales were considered for this study: (a) Autonomy (3 items; e.g., “*During this event I felt that my choices were based on my true interests and values*”), (b) Competence (3 items; e.g., “*During this event I felt that I was successfully completing tasks and projects*”) and (c) Relatedness (3 items; e.g., “*During this event I felt a sense of contact with people who care for me, and whom I care for*”). Each item was rated on a five-point Likert scale from 1 (totally disagree) to 5 (totally agree). Higher scores indicate a greater degree of perceived autonomy, competence and relatedness. All subscales showed good internal consistency (mean inter-item correlation was 0.640, 0.697 and 0.744 for Autonomy, Competence and Relatedness subscales, respectively).

#### 2.2.3. Attitudes toward COVID-19 Vaccines

Attitudes toward COVID-19 vaccines were evaluated with two subscales from the *Oxford COVID-19 Vaccine Confidence and Complacency Scale* [42]. The following subscales were considered for this study: (a) belief that the respondents may get COVID-19 and the vaccine will be effective (WRK subscale; 3 items; e.g., “*Do you think you will be infected with COVID-19 over the next months?*”) and (b) side effects (SE subscale; 3 items; “*The side effects for people from receiving the COVID-19 vaccine will be…*”). Each item was rated on a five-point Likert scale from 1 to 5, with item-specific response options [42]. Higher scores indicate a greater degree of negative attitude scores. Both subscales showed acceptable internal consistency (mean inter-item correlation was 0.165 and 0.354 for WRK and SE subscales, respectively).

#### 2.2.4. Pfizer-BioNTech and AstraZeneca VH

Vaccine hesitancy was evaluated with two questions (one for the Pfizer-BioNTech vaccine and one for the AstraZeneca vaccine) adapted from a previous study [39]. Participants were asked to rate their intentions to be vaccinated (“*Several COVID-19 vaccines are currently available. Are you intent on receiving the Pfizer-BioNTech/AstraZeneca COVID-19 vaccine*”?) on a 5-point Likert-type scale (1 = not at all, 2 = little, 3 = unsure, 4 = likely, 5 = very likely). A higher score indicated a lower level of hesitancy toward the COVID-19 vaccine. In order to evaluate the willingness to receive the vaccine, participants were classified as “vaccine-resistant” if they responded “*not at all*”, “vaccine-hesitant” if they responded “*little*” or “*unsure*”, and “vaccine-accepting” if they responded “*likely*” or “*very likely*”. For descriptive analyses, data about VH were treated as categorical, whereas for subsequent main analyses, data about VH were treated as continuous.

### 2.3. Statistical Analyses

As a preliminary step in the data analysis, the normality of continuous variables was checked; all variables had a normal distribution (−0.990 < Sk < 1.041; −1.410 < Ku < 1.282) [43]. There was no missing data on BPNs and ECs, attitudes toward COVID-19 vaccines and VH. Mean inter-item correlation was computed to assess internal consistency. Mean inter-item correlations between 0.15 and 0.50 indicate adequate internal consistency [44]. Descriptive statistics were used to describe the data based on frequencies and percentages for categorical data and mean and standard deviation for continuous data.

**Figure 1:**
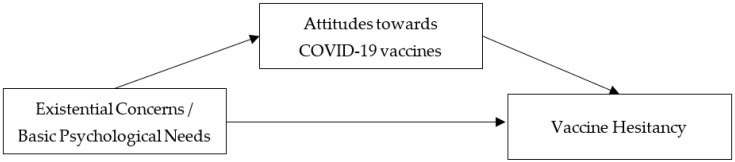
Theoretical model of the study.

The hypotheses of the study were tested using Structural Equation Modeling (SEM). The theoretical model tested in this study is displayed in Figure 1. Model testing was performed using Mplus software, v. 7.0. ECs were operationalized by three indicators: general existential concerns, death anxiety and avoidance. BPNs were operationalized by three indicators: autonomy, competence and relatedness. The overall goodness-of-model fit was assessed using the χ^2^ statistics (χ^2^/df ratios < 3 indicate reasonable fitting models), the comparative fit index (CFI, with values >0.90 indicating better fitting models) [45] and the root-mean-square error of approximation (RMSEA; values <0.08 indicate good fit) [46]. Subsequently, 95% confidence intervals (CIs) were computed using 5000 bootstrap resamples for indirect effects [47]. CIs that did not contain a zero value indicate a significant indirect effect.

### 2.4. Procedures

Respondents for this study were recruited using Clickworker. All respondents were living in Italy. At the end of the survey, they received 1 € as financial incentive. Data collection took place during the third wave of the COVID-19 pandemic in Italy (from April 2021 to May 2021). The questionnaire was anonymous, and at the end of the survey all participants received their payment from the platform. The online questionnaire took approximately 10–15 min to be completed. A pilot study was conducted with a group of participants (data excluded) to measure the internal consistency of the survey. The research was conducted in accordance with the ethical standards of the Italian Psychological Association (AIP), as well as the Declaration of Helsinki.

## 3. Results

### 3.1. Willingness to Receive COVID-19 Vaccine

Regarding the Pfizer-BioNTech COVID-19 vaccine, 29 participants (10.1%) were resistant, 64 (22.3%) were hesitant and 194 (67.6%) were acceptant. Regarding the AstraZeneca COVID-19 vaccine 94 participants (32.8%) were resistant, 92 (32.1%) were hesitant and 101 (35.2%) were acceptant. The observed differences in the acceptance of the two vaccine types were statistically significant (*p* < 0.001) for each category, with a small to medium effect size (φ = 0.480, 0.206 and 0.401 for resistant, hesitant, and acceptant, respectively).

### 3.2. Test of Hypotheses

**Figure 2:**
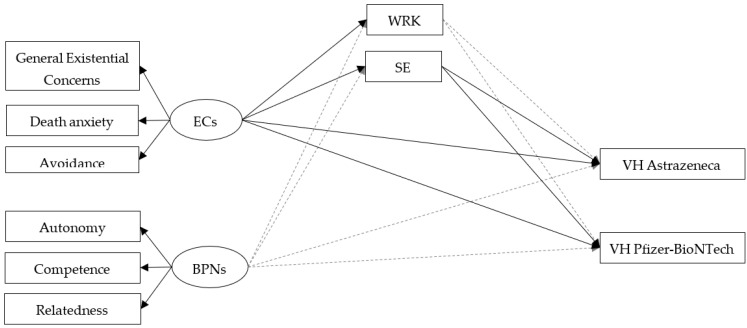
Structural equation modeling testing attitudes towards COVID-19 vaccines as a mediator of the relationship among ECs, BPNs and Pfizer-BioNTech and AstraZeneca VH. Note. ECs = Existential Concerns; BPNs = Basic Psychological Needs; WRK = Attitude toward COVID-19 vaccines—vaccine will be effective; SE = Attitude toward COVID-19 vaccines—side effects; VH = Vaccine Hesitancy; Errors and correlations were omitted from the diagram for clarity; Non-significant paths are represented by grey dashed lines; Significant paths are represented by black solid lines.

Descriptive statistics of the study variables are reported in Table 1. Table 2 and Figure 2 show results from the structural equation modeling. The model provided only a modest fit to the data (χ^2^ = 78.382, df = 25, χ^2^/df = 3.1, CFI = 0.945, RMSEA = 0.086, 95% RMSEA = 0.065 − 0.108) so the modification indices were used to improve the fit. More specifically, the model was modified by adding a covariance between WRK and SE subscales. This modification improved the model fit (χ^2^ = 43.633, df = 24, χ^2^/df = 1.8, CFI = 0.980, RMSEA = 0.053, 95% RMSEA = 0.027 − 0.078). The model accounted for 19% (R^2^ = 0.187) and 13% (R^2^ = 0.131) of the variance in Pfizer-BioNTech and AstraZeneca VH, respectively. As hypothesized, there were statistically significant positive associations between ECs and attitudes towards the COVID-19 vaccines (both WRK and SE subscales). Participants with higher scores on ECs also show higher negative attitudes towards COVID-19 vaccines. However, contrary to our hypothesis, no significant associations were found between BPNs and attitudes toward COVID-19 vaccines (neither for WRK nor SE subscales). Moreover, only the SE subscale, but not the WRK subscale, was related to Pfizer-BioNTech and AstraZeneca VH. Higher concerns about possible side effects of COVID-19 vaccines are related to lower levels of acceptance of vaccines, with almost the same magnitude between the two vaccine typologies. As previously hypothesized, mediation tests indicated that existential concerns (ECs) showed a significant indirect effect on Pfizer-BioNTech (standardized indirect effect value = −0.063, *p* < 0.05, 95% CI = −0.014/−0.003), as well as AstraZeneca (standardized indirect effect value = −0.059, *p* < 0.05, 95% CI = −0.015/−0.003) VH through SE, with almost the same magnitude between the two vaccine typologies. Finally, there was a statistically significant, albeit small, direct relationship between ECs and VH (both Pfizer-BioNTech and AstraZeneca). A totally mediated model without the direct effect between ECs and BPNs on Pfizer-BioNTech and AstraZeneca VH was estimated. The fit of this second model was compared to the first model by using the difference between the two model chi-squares [48]. The totally mediated model had an acceptable fit (χ^2^ = 53.523, df = 28, χ^2^/df = 1.9, CFI = 0.974, RMSEA = 0.056, 95% RMSEA = 0.033 − 0.079). The paths from ECs to WRK (β = 0.137) and SE (β = 0.162) subscales, as well as the paths from SE subscale to Pfizer-BioNTech (β = −0.378) and AstraZeneca (β = −0.336) VH were still significant. However, the chi-square difference (Δχ^2^ = 9.89, Δdf = 4) favored the partially mediated model.

## 4. Discussion

The present study examined both direct and indirect (through negative attitudes toward COVID-19 vaccine) relationships between ECs, BPNs and VH (both Pfizer-BioNTech and AstraZeneca). The study was conducted during the third Italian wave of the pandemic, when vaccines were just beginning to become widespread.

Globally, the results of the study showed that ECs demonstrate both a direct relationship with VH (for both Pfizer-BioNTech and AstraZeneca vaccines), as well as an indirect relationship through negative attitudes toward COVID-19 vaccines, due to their potential side effects. Surprisingly, the direct relationship was positive, i.e., individuals with higher ECs reported higher vaccine acceptance, whereas the indirect relationship was negative, i.e., individuals with higher ECs have lower vaccine acceptance, through increasing negative attitudes toward the possible side effects of COVID-19 vaccines.

Regarding the direct relationship between ECs and VH, our result is consistent with those of Mahmoodi and Peighambari [49] and Bodner et al. [50]. Moreover, previous studies showed that the pandemic is linked with increased ECs [33], which may afterwards affect health-related decisions about COVID-19, such as being vaccinated [32]. These results may be interpreted by referring to the dual-process of defense [51] incorporated in the Terror Management Health Model (TMHM) [30], which extends TMT to the domain of health behaviors. According to Pyszczynski et al. [52], consciousness of a possible danger or a death threat can be controlled by operating proximal defenses to decrease the feeling of vulnerability. Thus, proximal defenses may conduct people toward healthy choices such as engaging in healthy behavior or getting vaccinated in order to cope with existential fears and concerns [53].

Regarding the indirect relationship between ECs and VH, the results of the study suggest that individuals with greater fears linked to threats to human existence (ECs defined as general existential concerns, death anxiety and avoidance) also reported higher negative attitudes toward COVID-19 vaccines related to their efficacy and potential side effects. Moreover, participants with higher scores on negative attitudes (related to possible side effects of COVID-19 vaccines) were also less inclined to be vaccinated against COVID-19 (regardless of the type of vaccine). Thus, our results seem to confirm the mediating role of negative attitudes related to possible side effects of the COVID-19 vaccines in the relationship between ECs and VH. These findings are in line with previous research on VH. For example, previous studies showed that similar worries about flu vaccinations (e.g., about side effects, inefficiency, and general negative emotions) can lead toward VH, despite scientific evidence about their efficacy and safety [54]. From this perspective, Simione et al. [38] hypothesized that a belief in conspiracy theories could be a protective reaction against death anxiety. More recently, Palamenghi et al. [55] explained that a lack of faith in science may be an important factor in an individual’s low desire to receive vaccinations against COVID-19, thus justifying these results, which refer to the importance of the individual’s worries regarding the vaccination in encouraging this reluctance. Additionally, Walsh et al. [56] found that those who reported vaccine hesitancy were more likely to have more negative vaccination attitudes as well as perceiving higher risks associated with being vaccinated.

Thus, the two different underlying mechanisms identified in this study may be conceptualized as two different ways of reacting to fear provoked by the main threats to human existence. Similar results have also been found in previous studies on death anxiety, belief in conspiracy and vaccine hesitancy [38].

However, the results of the study did not confirm the mediating role of negative attitudes toward COVID-19 vaccine efficacy in the relationship between ECs and VH. During the third Italian wave of the pandemic, when COVID-19 vaccinations were quite novel, little information was available about the effectiveness of the vaccine, and there was a lot of confusion about the categories of people that could receive certain types of vaccine [57]. Moreover, COVID-19 vaccines have been targeted by the mass media and social media, which for a long time have been singling out news about the potential side effects of the COVID-19 vaccines (e.g., rare, severe thrombosis, mainly in young people, after inoculation with the AstraZeneca vaccine), and leading to individuals’ negative societal sentiment against them.

Finally, the results of the study showed that BPNs (i.e., autonomy, competence, and relatedness) were not related to VH neither directly nor indirectly (through negative attitudes toward COVID-19 vaccines). In other words, the assumption that individuals may react to experiences of need frustration with oppositional defiance [12,17] is not confirmed, even when attitudes towards the vaccine are introduced as mediators. This could be explained by the fact that VH is not linked to the cognitive aspects that refer to feelings of competence in the own activities, or to feel that the own activities are self-chosen and self-endorsed (autonomy), or feelings of closeness with some others (relatedness) [7]. These purely cognitive aspects probably do not have the same importance of existential concerns belonging to TMT in predicting individual levels of VH.

Overall, the results of the current study highlight the importance of examining psychological variables associated with vaccine hesitancy. Since the beginning of the pandemic and before the vaccination uptake, several studies have assessed beliefs and attitudes towards the COVID-19 vaccines, as well as some sociodemographic predictive factors of the individual willingness to get vaccinated. For example, Sherman et al. [58] found that increased inclination to accept COVID-19 vaccines was associated with older age, previous vaccination acceptance and positive vaccination beliefs and attitude. Paul et al. [59], in a cross-sectional survey, found that vaccine hesitancy was linked to female gender, having children and poor obedience to COVID government guidelines. However, limited research efforts have focused on more comprehensive psychological aspects. In this regard, Muldoon et al. [60] found that the reasons for vaccine hesitancy are linked to the fear of side effects and conspiracy beliefs, in accordance with our results. These findings suggest that vaccine hesitancy may not be entirely a problem related to a lack of knowledge, but it may be linked to the fear of unknown consequences. Greater science-supporting messages from experts about vaccine safety could be implemented to strengthen individual’s pro-vaccine evaluations and effectively address the emotional aspect of disinformation. As highlighted in past research, emotional engagement has been as successful as statistical information at warranting the good performance of health messages and people behavioral change [61], and it could be a suitable strategy in a public health campaign to promote COVID vaccination.

### 4.1. Limitations

Despite providing some significant findings in the scope of research into vaccination uptake, it is also important to acknowledge the presence of limitations within the study that should be considered for future. First, the results are based on a small sample recruited via a crowdsourcing platform, which cannot be considered representative of the Italian population. Second, the use of a single-item assessment tool limits us in drawing firm conclusions regarding vaccine hesitancy. However, a recent review showed important gaps in conceptualization and operationalization of vaccine hesitancy [62], and the development of a valid measure of vaccine hesitancy or resistance is strongly needed. Third, we evaluated individuals’ intention to be vaccinated and we do not know if an actual uptake followed this intention. Finally, a limitation concerns the age of the sample composed by adult population. Future studies could focus on age-stratified research to understand how beliefs such as attitudes might change and interact with the intention to get vaccinated, by age. Nevertheless, this research provides important evidence regarding the association between ECs and negative attitudes towards possible side effects of COVID-19 vaccines and VH among a population that has undertaken an extensive vaccination plan. The results of the study will serve as valuable information for government institutions in order to plan public health programs for vaccinating people worldwide in order to definitely defeat COVID-19.

### 4.2. Conclusions

The findings of the study highlighted that existential concerns can be related to Pfizer-BioNTech and AstraZeneca vaccine hesitancy. Thus, Terror Management Theory may be a useful approach to examine individuals’ COVID-19 health-related decisions. Further research is needed to increase individuals’ information, correct misunderstanding, address individuals’ doubt and fears, and foster positive attitudes toward COVID-19 vaccines [2,63].

## Figures and Tables

**Table 1:** Participants’ demographics and health-related data and descriptive statistics for the study variables.

	Sample (*n* = 287)
Age, M (SD)	36.04 (12.07)
Gender, *n* (%) females	172 (59.9)
Educational level, *n* (%)	
13 years of schooling	134 (46.7)
Degree/post-graduate	149 (51.9)
missing	4 (1.4)
Italian regions, *n* (%)	
Northern	99 (34.5)
Central	48 (16.7)
Southern	125 (43.6)
missing	15 (5.2)
Pathologies, *n* (%) yes	35 (12.2)
Own diagnosis of COVID-19, *n* (%) yes	20 (7.0)
COVID-19 among relatives, *n* (%) yes	140 (48.8)
ECs—General Existential Concerns, M (SD)	30.71 (11.41)
ECs—Death Anxiety, M (SD)	15.57 (6.35)
ECs—Avoidance, M (SD)	9.45 (3.95)
BPNs—Autonomy, M (SD)	9.42 (3.13)
BPNs—Competence, M (SD)	9.59 (3.17)
BPNs—Relatedness, M (SD)	10.01 (3.17)
Attitudes toward vaccine—WRK, M (SD)	6.73 (2.22)
Attitudes toward vaccine—SE, M (SD)	7.73 (4.26)
VH—Pfizer-BioNTech, M (SD)	3.88 (1.31)
VH—AstraZeneca, M (SD)	2.72 (1.50)

Note: ECs = Existential concerns; BPNs = Basic Psychological Needs; WRK = Attitude toward COVID-19 vaccines—vaccine will be effective; SE = Attitude toward COVID-19 vaccines—side effects; VH = Vaccine Hesitancy.

**Table 2:** Parameter estimates from the structural equation modeling for existential concerns, basic psychological needs, attitudes toward COVID-19 vaccines and vaccine hesitancy.

Model	β	SE	*p*-Value	95% CI
Direct paths				
ECs → WRK	0.137	0.015	0.047	0.005/0.054
ECs → SE	0.161	0.027	0.015	0.022/0.112
BPNs → WRK	0.094	0.075	0.226	−0.032/0.213
BPNs → SE	−0.085	0.138	0.254	−0.383/0.066
ECs → VH AstraZeneca	0.132	0.009	0.045	0.003/0.034
ECs → VH Pfizer-BioNTech	0.138	0.007	0.017	0.006/0.030
BPNs → VH AstraZeneca	−0.064	0.045	0.353	−0.116/0.029
BPNs → VH Pfizer-BioNTech	0.040	0.036	0.523	−0.036/0.082
WRK → VH AstraZeneca	0.024	0.041	0.700	−0.055/0.081
WRK → VH Pfizer-BioNTech	−0.094	0.043	0.192	−0.127/0.012
SE → VH AstraZeneca	−0.366	0.018	0.000	−0.158/−0.098
SE → VH Pfizer-BioNTech	−0.392	0.018	0.000	−0.150/−0.090
Indirect paths				
ECs → WRK → VH AstraZeneca	0.003	0.010	0.737	−0.013/0.019
ECs → WRK → VH Pfizer-BioNTech	−0.013	0.013	0.318	−0.034 /0.008
ECs → SE → VH AstraZeneca	−0.059	0.026	0.021	−0.101/−0.017
ECs → SE → VH Pfizer-BioNTech	−0.063	0.028	0.025	−0.110/−0.017
BPNs → WRK → VH AstraZeneca	0.002	0.008	0.777	−0.011/0.015
BPNs → WRK → VH Pfizer-BioNTech	−0.009	0.012	0.446	−0.028/0.010
BPNs → SE → VH AstraZeneca	0.031	0.028	0.259	−0.014/0.077
BPNs → SE → VH Pfizer-BioNTech	0.033	0.030	0.258	−0.015/0.082
Correlations				
WRK with SE	0.339	0.577	0.000	2.139/4.010
VH AstraZeneca with VH Pfizer-BioNTech	0.438	0.096	0.000	0.549/0.869

Note. ECs = Existential Concerns; BPNs = Basic Psychological Needs; WRK = Attitude toward COVID-19 vaccines—vaccine will be effective; SE = Attitude toward COVID-19 vaccines—side effects; VH = Vaccine Hesitancy.

## Data Availability

Data available on request from the authors.
